# Supplementary material for: Are carbon emissions trading and green financial instruments synergistic? -Comprehensive quantitative research based on content analysis
Source: PLoS One. 2024 Mar 7;19(3):e0298601. doi: 10.1371/journal.pone.0298601 (PMC10919601; doi:10.1371/journal.pone.0298601)
Supplement: S1 Data — (ZIP) [file pone.0298601.s001.zip › Data/地级市Z_F工作报告.zip!/╜¡╬≈/╛┼╜¡╩╨/└┤╫╘╣1⁄2╓┌║┼í╛┬φ┐╦╩2╛▌═°í┐_2006.docx]

各位代表：

现在，我代表市人民政府向大会作工作报告，并就《九江市国民经济和社会发展第十一个五年规划纲要（草案）》作出说明，请予审议，请政协委员和其他列席会议的同志提出意见。

一、“十五”及２００５年工作回顾

“十五”时期，是我市遭受百年未遇特大洪灾之后的恢复发展期。五年来，在省委、省政府和市委的正确领导下，全市广大干部群众深入贯彻党的十六大精神，与时俱进，奋力拼搏，克服了国际国内宏观环境变化带来的困难，战胜了非典、台风、地震等多种自然灾害，完成或超额完成了“十五”计划的各项任务。

国民经济持续快速增长，综合实力明显增强。全市生产总值由２０００年的２１３．１２亿元增加到２００５年的４２８．９２亿元，年均递增１３．６％，比“十五”计划目标３５９．１亿元超过６９．８２亿元。财政总收入由２０００年的２０．５０亿元增加到２００５年的３９．８１亿元，年均递增１４．２％，比“十五”计划目标３３．０２亿元超过６．７９亿元；地方一般预算收入由２０００年的１０．５亿元增加到２００５年的２２．４５亿元，年均递增１６．４％。县区财政收入提前一年实现翻番。三次产业结构由“九五”期末的２０．２：４５．０：３４．８调整为１６．８：５０．１：３３．１。养殖业产值超过种植业产值，经济作物面积超过粮食作物面积；培育国家和省级农业龙头企业１３家，创绿色食品品牌４６个，建立省级无公害农产品基地７１个；农民人均纯收入由２０００年的１８５１元增加到２００５年的３２６９元，年均递增１２．０％，比“十五”计划目标２４９０元超过７７９元。培植利税超亿元企业５户，石油、化工、建材、纺织成为全省最大的产业集群。全市１２个工业园入园企业年均递增４６．７％，园区经济已占全市工业总量四分之一。２００３年规模以上工业整体扭亏，２００４年规模以上工业增加值、销售收入和利税实现了“三年翻番”。市场建设步伐加快，五年培育成交额超１０亿元的市场２个。

改革开放深入推进，开放型经济成为推动发展的主导力量。出台了深化改革、扩大开放的６０条措施，大力推进国有企业改革，市属企业改制完成５２户，县属企业改制基本完成。精简市级机构１５个，撤乡并镇４５个，９０％的事业单位和人员实行合同聘任制。全面推进农村税费改革，实现了农民合同内零负担。林权制度改革顺利实施，公共财政体制建设迈出重要步伐，共青管理体制基本理顺，卫生、教育、公路、粮食、住房、电力、科技等体制改革稳步推进。开放型经济成效显著，实际利用外资由２０００年的３０００万美元增加到２００５年的３．２３亿美元，年均递增６０．８％，五年累计完成１２．０６亿美元，比“十五”计划目标４亿美元超过８．０６亿美元；实际利用内资由２０００年的１７．８亿元增加到２００５年的１３２．６０亿元。出口总额由２０００年的３７８９万美元增加到２００５年的１．１８亿美元，年均递增２５．４％，五年累计完成３．３１亿美元，比“十五”计划目标２．５亿美元超过０．８１亿美元。外贸国际集装箱吞吐量由２０００年的４０００标箱增加到４．６万标箱，增长了１０倍多。吸引了日本铃木、新加坡金鹰、台湾远东、香港华孚、中国五矿、３Ｔ光电等一批知名企业落户九江。开放型经济对经济增长的贡献率为６２．６％，占财政收入的比重为６０％。检验检疫、海关、海事、外汇管理等驻市机构和外事、侨务、对台事务、驻外机构在对外开放中发挥了重要作用。五年来，九江知名度不断提升，先后荣获“中国优秀旅游城市”、“全国双拥模范城”、“中国魅力城市”等桂冠，庐山被评为世界地质公园。接待境外旅游人数由２０００年的３．０８万人次增加到２００５年的８．０２万人次，年均递增２１．１％；旅游外汇收入由２０００年的７０２．１０万美元增加到２００５年的２３５７．４５万美元，年均递增２７．４％，旅游经济总量五年翻番。

投资结构发生重大变化，经济自主增长机制逐步形成。这五年是我市投资主体变化最大、投资规模扩张最快时期。全市固定资产投资由２０００年的４４．７７亿元增加到２００５年的１９０．２０亿元，年均递增３３．６％；五年累计完成固定资产投资５７８．６３亿元，比“十五”计划目标２５０亿元超过３２８．６３亿元。二产投资占总投资的５０％，农村基础设施、公共卫生、基础教育等方面的投资进一步加大。运用ＢＯＴ方式建设老鹳塘污水处理厂，成立鄱湖水产股份有限公司，拓展了新的融资渠道。民营企业户数年均增长２３．９％，注册资本年均增长４９．８％，上缴税金年均增长３７．５％。民间投资在固定资产投资中的比重由１０．２％上升到５０．７％。利用内外资总量占全社会投资的比重达到６６％，投资多元化趋势明显，标志着我市经济自主增长机制逐步形成。

基础设施不断完善，城乡面貌发生较大变化。２８２．９公里江湖大堤全面整治，移民建镇工程全面完工，安置沿江滨湖移民４５万人。累计治理各类水库１５４座，综合治理水流域１１１２平方公里，建设国家级重点生态功能区１个，生态示范区２个，各级自然保护区２４个。累计完成公路建设６３４４公里，环庐山、修武南线等公路和九武铁路复线建成通车。电力总装机容量从９６万千瓦提高到２００万千瓦，农村电网改造全面完成，实现了城乡同网同价。市区基础设施建设累计投资１８亿元，新增市区道路１５０万平方米，新增绿地１８６万平方米，新建了九威大道、长江大道等１０多条交通主干道，我市被评为全省第一个国家级“畅通工程二等城市”，全面完成《九江市总体规划》修编工作，白水湖公园、滨江生态带等一批园林绿地的建成，两湖治理，环湖的美化、亮化提升了城市品位。各县（市、区）城镇建设步伐加快，城区框架拉开，特色明显。

社会各项事业协调发展，人民生活水平不断提高。科技成果转化率比“九五”期末提高了１０．２个百分点，累计取得国际领先水平科技成果５项，国际先进水平２０项，获得国家科技进步奖２项。完成教育投资５６．２亿元，新增校舍８０．７万平方米，改造危房６１万平方米，完成企业学校剥离和民师转编工作，全市“两基”工作全面达标，减免贫困学生学杂费８５００万元，民办学校新增９８３所，九江学院的组建结束了我市没有本科院校的历史。完成了第三次文物普查，修缮恢复了锁江楼、濂溪景区等一批历史文物。基本实现行政村广播电视“村村通”。加大卫生防疫事业的投入，全市疫情报告网络基本建立，健全了疾病控制和医疗救治体系，五年查螺灭螺７９．８万亩，免费救治晚血病人１２７０例，结核病人８７９５例。积极实施全民健身计划纲要，竞技体育跻身全省前列，荣获全国群众体育工作先进单位。累计发放农村“五保户”供养补助资金７６４０万元，集中供养率由“九五”期末的１８．８％提高到７０％。养老保险、失业保险、医疗保险覆盖面扩大，社保金发放率达１００％。累计新增就业人员２０．９万人，下岗再就业９．８万人，困难群体再就业２万人，城镇登记失业率４％。筹措资金１．１３亿元，安置二次搬迁库区移民和三峡移民６５００人，安置深山移民４２００户，解决了７．０９万贫困人口温饱。

城镇居民人均可支配收入由２０００年的５０８１元增加到２００５年的８７１３元，年均递增１１．４％，比“十五”计划目标６７００元超过２０１３元。市区居民人均居住面积达２０．９平方米，比“九五”期末增加７．７平方米。全市本外币各项存款余额由２０００年末的１６３．１亿元增加到２００５年末的３７４．１亿元，增长１．３倍。档案、残联、红十字会、民族宗教、老龄事业又有新起色，防汛抗旱、气象、水文、地震、地质、人防、森林防火等单位在防灾减灾中发挥了重要作用。粮食、物资、供销、医药、盐业、烟草等行业在服务九江经济建设和人民生活方面作出了新的贡献。

经济发展环境有较大改善，民主法治进程加快。从机制、体制层面进一步规范政府行为，健全土地出让、工程招投标、政府采购、城市管理、应急体系建设等制度。全面开展经济发展环境整治工作，推行行政职能部门公开承诺制，成立了“行政服务中心”，建立外商投资“绿色通道”，取消行政审批事项８９５项，开通了“经济１１０”，开展执法监察和行风评议活动，加大对影响投资环境和损害群众利益案件的查处力度，查出违法违纪金额７０５３万元。统计、审计、工商、国税、地税、物价、安监、质监、药监等部门在规范市场经济秩序中做出了新贡献。全力打造“开拓、透明、高效、诚信”政府，累计出台规范性文件９６个，在全省率先建立政府信息公开制度，“中国九江”网站在宣传九江中发挥了重要作用。圆满完成“四五”普法，认真贯彻《行政许可法》，累计办理行政复议４５６件。依法接受人大法律监督和工作监督，充分发挥政协的政治协商、民主监督和参政议政作用，认真办理各级人大代表议案和政协委员提案。建立领导包案责任制和信访责任追究制度，促进信访突出问题的解决。加大社会治安综合治理力度，严厉打击各类刑事犯罪?熏铲除了一批涉黑涉恶势力?熏查禁“毒赌色”违法犯罪活动，全力维护社会政治稳定和治安秩序平稳。加强国防教育和后备力量建设，驻浔部队、武警官兵在急难险重的关键时刻发挥了重要作用。

２００５年，在市委坚强领导下，我们乘“两新”大讨论的东风，积极而强劲地推进“３＋１”发展战略，努力排除宏观环境偏紧的困境，沉着应对台风、地震等突发灾害，众志成城，加快发展，形成了经济和社会发展的强劲势头，荣登中国城市综合实力百强第７５位，朝着建设美丽、富裕、和谐、强盛的社会主义新九江迈出了坚实的第一步。

经济总量实现突破性增长。全市生产总值比上年增长１５．４％，是十一年来最高增幅；财政总收入增长１３．５％，地方财政一般预算收入增长１９．４％；固定资产投资增长３２．６％，高于全省平均水平；社会消费品零售总额增长１４．０％；城镇居民人均可支配收入增长１１．４％；农民人均纯收入增长１２．７％，自’９８洪灾之后首次超过全省平均水平。

以大开放为总纲成效显著。全市共引进５００万元以上工业项目１１３２个，其中超亿元工业项目２１个，实际进资增长２６．２％，新批外资项目１１１个，实际利用外资增长４．８％。外贸出口首次突破１亿美元，增长３５．６％。新增４０家备案登记进出口权企业。成功申报国家级出口加工区，填补了江西省的空白，封关前的各项准备工作进展顺利。

推进大工业再创历史新高。５２个重大工业项目累计完成投资７９．５亿元，３Ｔ光电、绿冬丝科、华林特种钢等３０个项目竣工，１０万吨有机硅、４６００吨气相二氧化硅等项目开工建设。全市规模以上工业企业完成增加值９０．３９亿元，增长２６％；完成销售收入３３０．４３亿元，增长２６．３％；实现利税２４．６４亿元，增长４２．５１％。在重点骨干企业效益下滑的情况下，实现工业利润８．０９亿元，增长２．８倍，工业经济效益综合指数为１３４．４。

构建大旅游格局有了良好开端。编制完成九江旅游业发展规划，全市旅游规划体系逐步完善。庐山管理体制改革取得实质性进展，被评为全国文明风景旅游区，国务院批准了云居山－柘林湖为国家重点风景区。全市接待入境旅游者增长３２．７％，旅游外汇收入增长４１．８％，接待国内旅游者增长２２．８％，国内旅游收入增长２４％，旅游总收入增长２４．４％。

大物流建设迈出大步伐。启动物流园区规划，加大九江港扩建力度，打通疏港道路，九江国际水运中心集装箱吞吐能力增至１０万标箱，湖口国际集装箱码头前期工程进展顺利。十里服装市场建成营业，九九商城、天马建材城等市场扩建工程基本完工。全市注册登记物流企业４５３户。

城市大建设亮点纷呈。形成了城市规划体系，城市规划区建设用地控制性规划覆盖率达９２％；实现土地资产收益６．３亿元。三水厂二期扩建、青年南路、赣北地震监测中心、沙河水厂改造等一批工程竣工。白水明珠、南湖市民文化广场、龙开故道、胜利公园、体育中心、老年活动中心、疾控中心、少儿活动中心、廉租房小区、胜利大道、长虹西路、日元贷款城市防洪工程等４０个项目顺利实施。深入开展市容市貌、环境卫生、广告管理、城市违章建筑的整治活动，完成１２０多条边街小巷整治，改造人行道１５万平方米，城区亮化设施增加了２倍，创建园林城市通过省级验收。

实施“三百工程”扎实有效。１００个重大项目累计完成投资１２１．１７亿元，昌铃汽车、铃木发动机、仪长原油管道九江段等４１个项目竣工，第一辆利亚纳轿车下线圆了九江人的汽车梦。铜九铁路、亚泥三期等５９个项目正在加紧建设。１００个示范村庄建设投入资金３４３９万元，新修通村公路和硬化路面２７８公里，改水改厕４４６２户，新建沼气池１７６０户，新建文化场所７６个，完成了示范村庄规划，“一村一品”产业格局正在形成，实现了一年有看得见变化的目标。组织全市７０多个单位、社会团体１．６万人实施１００件惠民实事，投入资金１１．６７亿元，惠及群众５２０多万人次，解决了新生自带课桌椅问题，农村远程教育工程基本完工，建设残疾人康复中心、百所福利院、敬老院。

抗灾救灾经受了严峻考验。去年是多灾之年，数灾并发，台风、暴雨、病虫害等灾害对我市经济社会和农民增收产生了很大影响。面对接踵而至的自然灾害，我们迅速反应，沉着应对，果断决策，科学处置，将灾害损失降到最低限度。尤其是“１１·２６”地震发生后，在党中央、国务院的亲切关怀下，在中央各部委和全国各地大力支持下，在省委、省政府和市委直接领导下，迅速启动应急预案，立即开展抗震救灾，适时转入灾后重建工作。各级领导干部靠前指挥，受灾群众积极开展生产自救，社会各界无私援助，及时妥善安置灾民和救治受伤人员，加强灾区物资供应和卫生防疫，保障灾民基本生活和灾区社会稳定。多渠道筹集资金，全面开展灾后重建工作，民房、学校、医院、敬老院重建和震损水利工程除险加固工作正在抓紧进行。

各位代表，回顾五年发展历程及去年工作，成绩来之不易，是省委、省政府和市委正确领导，市人大依法监督和市政协参政议政，全市人民和所有在浔工作的同志们、朋友们辛勤劳动的结果。我谨代表市人民政府，向全市广大工人、农民、知识分子、干部和离退休老同志，向各民主党派、工商联和社会各界人士，向驻浔解放军、武警官兵和公安干警，向所有参与、关心和支持九江发展的海内外朋友表示衷心的感谢并致以崇高的敬意！

我市现代化建设取得令人鼓舞的成绩，但发展中也存在一些矛盾和问题，主要是：经济总量偏小，经济实力不强；自主创新能力较弱，经济增长方式比较粗放；中心城市集聚辐射和带动能力不强；影响经济发展的体制性、机制性障碍还较为突出，经济社会发展环境有待进一步优化；农民持续增收难度大；就业和再就业压力比较大；影响社会和谐稳定的因素依然存在；政府依法行政能力和效能建设有待进一步提高。对于前进中的问题，我们一定高度重视，在今后的工作中努力加以解决。

二、“十一五”经济社会发展主要任务及２００６年工作

各位代表，我市人均生产总值已经突破１０００美元，标志着全市经济社会发展跨入了一个新的历史阶段。今后五年是我市加快发展、实现赶超的关键时期。根据市委《“十一五”规划建议》，我们制定了《国民经济和社会发展第十一个五年规划纲要（草案）》，提请大会审议。坚持以科学发展观统领全市经济社会发展全局是《纲要》最鲜明的特点，建设美丽、富裕、和谐、强盛的社会主义新九江是“十一五”时期九江发展的基本目标，以大开放为纲、全力推进“３＋１”是“十一五”时期建设新九江的重大战略，扩张经济总量、壮大经济实力是“十一五”时期建设新九江的主要任务，提高绝大多数人民根本利益实现度是“十一五”时期建设新九江的落脚点。

未来五年，政府工作总的要求是：坚持以邓小平理论和“三个代表”重要思想为指导，以科学发展观统领经济和社会发展全局，紧紧围绕市委提出的指导思想和总体要求，高扬“和谐”、“发展”两面旗帜，坚定不移地实施以大开放为纲，全力推进大工业、大旅游、大物流和城市大建设的发展战略，繁荣社会各项事业，努力建设美丽、富裕、和谐、强盛的社会主义新九江。

未来五年，我市国民经济和社会发展主要预期目标是：全市生产总值年均增长１４％，实现经济总量翻一番，力争人均生产总值翻一番。财政总收入年均增长１６％，固定资产投资年均增长３０％，工业化率４８％，城镇化率５０％，城镇居民人均可支配收入年均增长１０％，农民人均纯收入增长１０％，社会消费品零售总额年均增长１４％，城镇登记失业率控制在４％以内，人口自然增长率控制在８‰以内，单位生产总值能耗下降２０％。未来五年，我们的工作任务是：

努力建设一个山川更加秀美，人与自然更加和谐的新九江。充分保护好九江原汁原味的生态环境和自然景观，继续推进天然林保护、退耕还林、水土流失治理和湿地保护等生态工程，全市城镇绿化率达到４０％，中心城市每年新增绿地６０万平方米，人均公共绿地达到８平方米以上，净增森林面积１５０万亩，森林覆盖率达到５２％，治理水土流失面积１５０万亩，工业固体废弃物综合处理率达到９６％，中心城市生活污水集中处理率超过７０％，生活垃圾无害化处理率达到１００％，中心城市空气质量达到国家二级标准。

努力建设一个城乡更加富庶，人民生活更加殷实的新九江。按照“最适宜人类居住”的现代化山水生态港口旅游城市的定位，争取到２０１０年左右把九江建成市区人口过百万的区域中心城市。高起点规划，高质量建设，高标准管理，把九江打造成基础设施配套，功能完备，品位高雅，与自然融会贯通的中国生态名城，创建全国文明卫生城。进一步消除城乡二元结构的体制性障碍，加快县城和中心集镇发展，重点建设好２０个中心集镇。充分发挥农民的主体作用，在继续推进“示范村庄”建设的基础上，广泛开展社会主义新农村建设。城镇人均居住面积达到３０平方米，农村人均居住面积达到４０平方米。城乡居民恩格尔系数分别降到３５％和４５％以下。

努力建设一个社会更加和睦，民风更为纯朴的新九江。坚持在和谐中求发展，以发展促和谐。不断完善社会突发事件应急管理体系，提高处理各种突发事件能力。巩固和提高义务教育水平，农村义务教育阶段家庭困难学生均能享受“二免一补”，基本消除中小学危房，基本普及高中阶段教育，大力发展高等教育和职业技术教育，高等教育毛入学率达到２５％。加快建设覆盖城乡的新型社会救助体系，开展农村最低生活保障制度试点，有效保障农村“五保”对象、城镇“三无”人员以及失地农民的基本生活。加快社区卫生服务体系建设，力争卫生服务人口覆盖率达９５％以上。完成２０户以上的自然村广播电视村村通工程，重点解决农村６５万人口饮水安全问题，建设乡村公路５４００公里。

努力建设一个财力日见充裕，实力日益壮大的新九江。狠抓重大项目建设，坚持大招商，招大商，着力培植主体财源，特别是壮大市本级财源。培育石油化工、机械电子、冶金建材、纺织服装、电力能源、食品加工等优势产业，提升传统产业，发展高新技术产业，大力开展自主创新，鼓励企业走名牌发展之路。加快昌九工业走廊、长江沿岸开发，全力打造“Ｔ”型工业布局，工业增加值年均增长２１％，工业经济总量实现恢复性进位。进一步扩大对外开放，实际利用外资年均增长１５％，外贸出口年均增长２５％。加快县域经济发展，培植财政收入超５亿元的县２个，超３亿元的县５个。

“十一五”期间，我们要牢牢把握以下几点：

牢牢把握发展这一永恒主题，坚定不移地把发展作为第一要务，切实解决我市经济总量不大的主要矛盾。作为一个经济欠发达地区，扩张总量是当务之急。目前我市经济和社会发展还面临着许多难题，建设资金不足，人才严重匮乏，人民生活水平有待提高，生活环境需要改善，城市要做大做强，下岗职工要就业，社会保障体系要健全等等，这些问题的解决都有赖于财政收入的增长和经济实力的增强，发展是解决所有问题的关键。目前，九江无论与沿江同类城市比，还是与省内兄弟城市比，最大的差距，就是经济总量的差距，因此，必须紧紧抓住扩张经济总量这个主要矛盾，坚持以大开放为纲，强力推进“３＋１”战略，集中力量抓大事，统筹兼顾保重点，在提高经济质的基础上，努力实现量的新扩张。

牢牢把握转变经济增长方式这个重点，坚定不移地把自主创新贯穿于“十一五”全过程，为经济和社会发展提供强大动力。在国家实施宏观调控的大背景下，我市土地、电力、能源等资源的承载能力面临巨大压力，必须坚持把经济效益、社会效益、生态效益三者有机结合起来。建设创新型九江，把增强自主创新能力作为转变经济增长方式的中心环节，加快形成有利于自主创新的体制机制环境，增强我市各行各业的自主创新能力。继续深化经济和行政管理体制改革，充分发挥市场在资源配置中的基础性作用。“既要金山银山，更要绿水青山”，力促经济增长由粗放型向集约型转变，使经济社会发展与资源、环境相适应，着力提高资源利用效率，降低物质消耗，保护生态发展，构建节约型社会、环境友好型社会。

牢牢把握构建和谐社会这个根本，坚定不移地贯彻执政为民思想，切实维护人民群众的根本利益。坚持以人为本，始终做到维护群众的利益高于一切，关心群众的疾苦重于一切，解决群众的困难先于一切，倾注真情、竭尽全力、真心实意办一些顺民心的实事、得民心的好事、暖民心的难事，最大限度地满足人民群众日益增长的物质文化需要，突出解决好人民群众最关心的就业、社保、扶贫、教育、医疗、环保和安全等问题，营造人民群众安居乐业的良好环境。

２００６年是实施“十一五”规划的开局之年，也是建设新九江的奠基之年，我们将紧紧围绕市委提出的“一个中心、两个根本点”工作重点，扎实推进“３＋１”发展战略，加快社会主义新农村建设步伐，切实提高政府执行力，努力提高群众满意度，全面加强社会主义经济建设、政治建设、文化建设和社会建设，促进经济既快又好发展和社会全面进步。今年全市经济和社会发展的主要预期目标是：全市生产总值比上年增长１５．０％，财政总收入增长１５．０％，地方一般预算收入增长１６．０％，实际利用外资增长１５．０％?熏引进内资增长２０．０％，规模以上工业增加值增长２０．０％，固定资产投资增长２５．０％，城镇居民人均可支配收入增长１０．０％，农民人均纯收入增长９．０％，城镇登记失业率控制在４．０％以内，人口自然增长率控制在７．７３‰以内。为实现上述目标，今年重点抓好八个方面的工作：

（一）以建设社会主义新农村统揽农业农村工作，改善农民生产生活条件，增加农民收入

建设社会主义新农村。积累反哺农业实力，建立反哺机制，按照中央提出“生产发展、生活宽裕、乡风文明、村容整洁、管理民主”的要求，认真总结１００个示范村庄建设经验，进一步探索建设社会主义新农村的途径。完成农村公路建设１０００公里，砂石路２００公里，加快农村教育、文化、卫生等社会服务设施建设，移动通信实现村村通，联通通信实现乡乡通。确保完成３５座省补助重点小（一）型病险水库除险加固。推进改水改厕改线和“四旁植树”，新建沼气池２．５万户以上。重点加强对林业资源和水资源的保护，完成水土流失综合防治面积３０万亩，解决农村１万人口饮水安全。全力以赴抓好地震灾区的住房、学校、医院、敬老院和震损水利工程的重建。

推进现代农业建设。进一步优化农业产业结构，在稳定粮食、棉花等常规农业生产的基础上，大力发展特色经济作物、畜牧业和水产业的生产规模，扶植鄱湖水产、美庐乳业等龙头企业，完善“企业＋农户”的利益联结机制，突出发展水产、茶叶、果业、花卉苗木等主导产业和产业基地。降低准入门槛，鼓励各种资本参与农业开发经营。加强农村专业合作经济组织建设，培育各类专业经纪人队伍。

增强农民增收后劲。加大农业科技投入，力促农业标准化有新的突破。大力推广良种、良法，加快先进实用技术应用普及。完成８万亩中低产田改造任务。建立开放式培训机制，发挥各类培训阵地作用，培训农民工２万人以上。认真做好以乡村最低生活保障、低保家庭子女免费入学、孤寡老人集中供养、农村新型合作医疗、被征地农民基本生活保障为内容的农村“新五保”工作。千方百计筹措扶贫资金，更加注重扶贫开发实效。进一步规范涉农规费，防止农民负担反弹。精心组织实施第二次农业普查。

（二）以对外开放为总纲，提高招商选资的质量和水平

亲商安商造氛围。确立“三分招商，七分安商”理念，进一步健全优惠政策的制度化和法制化，保持招商政策、措施的连续性和稳定性。建立招商、建设、投产全过程服务体系，落实帮扶责任制，在登记注册、土地审批、水电安装、融资协调等方面给予优质服务，切实解决客商实际困难。继续发挥经济１１０和外商投诉中心的作用，完善外商投诉月报制和客商联系卡制度，形成随时受理、及时办结机制。

搭建平台求实效。以昌九工业走廊和沿江产业带为平台，利用特色资源，精心包装推介一批具有竞争力的招商项目，吸引客商投资落户。打造对接通道优势，为机场复航、铜九铁路、昌九城际铁路、武吉高速、九瑞高速、长江二桥等重点工程提供有力的服务保障。把长珠闽作为承接产业转移重点，主攻世界５００强、国内大集团和民营１００强企业的引进。继续办好赣台（九江）经贸合作研讨会，荷兰、新加坡和浙江、福建、上海等地的招商活动。进一步发挥福建商会、浙江商会、广东商会、台商协会在招商引资中出谋划策、牵线搭桥的作用。

主攻项目再升温。实现由比拼优惠政策为主向比拼优势为主转变；由开展活动为主向紧密联系客商为主转变；由项目签约为主向实际进资开工建设为主转变。充分发挥我市优势产业和核心企业的作用，结合建材、石化、纺织等支柱产业的做大做强，实行产业链和外商熟悉的方式招商。对招商项目进行认真梳理，区别分类，确定专人，限定时间，紧密联系１００个以上重点客商，加强调度，力争落户１００个重大项目。

（三）加大投入，做实园区，以重大项目推动大工业

大上项目扩总量。力争新时代高新能源、台湾冠贺运动器材、远东纺织、挪宝电器、４０万吨硫酸、凤竹纺织等项目开工建设。力促环鄱阳湖地区风力发电项目列入国家“十一五”规划，抓好核电一期建设的前期工作。各县（市、区）选择１－２个具有潜在优势的产业，引进一批具有较强带动能力的龙头企业，培育特色产业集群。

做实园区扩总量。加快出口加工区建设进度，确保３月底封关验收。加快码头、沙河、金砂湾３个工业城和１２个工业园区水、电、路等基础设施建设步伐。鼓励边远县在九江开发区内异地开发，自行建园。扶植沿路沿江县（市、区）工业园建设，着力提高工业园区产业关联度，延长产业链，促使产业向园区集群，项目向园区集中，客商向园区集聚，使园区成为我市工业发展的主引擎。力争规模以上工业增加值达到１２６亿元。

狠抓达标扩总量。继续做好５２个重大工业项目的后续工作，全力抓好昌铃汽车及发动机一期项目的达产达标，推动石化１００万吨焦化装置和１２０万吨加氢装置、星火１０万吨有机硅、巨石３万吨池窑拉丝、卡博特４６００吨气相二氧化硅项目尽快竣工投产，加快亚泥３００万吨水泥熟料、赛得利６万吨粘胶短纤、新康达３万吨离子膜烧碱等项目的建设步伐。抓好奥盛、七一三、华孚等一批企业的技术改造。加大对骨干企业的扶持力度，力争新增利税超亿元企业２－３个。

盘活存量扩总量。进一步加快国有企业改革步伐，建立健全国有资产保值增值责任考核体系。以国有企业改制为突破口，采取靠大联大、破产重组等多种形式，盘活国有存量资产，用存量换取增量，继续抓好江西制氧机厂、原九江啤酒厂、原江洲造船厂等１０户企业改制和资产重组。

（四）以平台建设为重点，加快港口建设，壮大物流企业

培育物流平台。充分利用长江黄金水道和四省毗邻的优势，发挥九江国家一类口岸的作用，加大物流平台建设力度。规划启动以集装箱转运为主的湖口物流园区，以仓储、配送、交易为主的九江综合物流园区，以服务运输、仓储为主的官湖物流园区的建设。加快昌铃汽车、石化产品、邮政集散等十大物流中心的建设步伐。进一步培育壮大京九农副产品中心批发市场、华东装饰材料市场等十大市场。建立市、县两级物流行业协会，引进和培养物流人才。

发展临港产业。抓住国家开发黄金水道的契机，充分利用并保护好沿江岸线资源，调整港口结构，发展公用码头。加大老港区的扩改力度，新增散货通过能力６０万吨，集装箱吞吐量达６万标箱。加速湖口国际集装箱码头建设，适度超前、适时启动官湖港区。实行工港、工贸联动，促成铁路、公路、水运、空运等各种运输方式的有效衔接，提高九江接收长江和京九双向物流的能力，增强对四省毗邻地区的辐射力。

壮大物流企业。进一步开放物资贸易和运输服务市场，为物流业发展提供宽松的外部环境。支持大型连锁、批发代理、第三方物流和大型工业企业开展物流配送，凡具备条件在九江创办物流企业，将在注册登记、经营项目审批等方面简化手续，提供方便，对符合条件的物流企业可申请设立海关监管。鼓励各种资本进入物流市场，放宽资本限制，提高审批透明度，大力引进国内外物流企业入驻我市。

（五）积极理顺体制，全面整合资源，构建大旅游格局

打造旅游核心品牌。培育庐山、柘林湖、市区、共青、温泉和鄱阳湖等六大景区，依法依规加快旅游景点的开发和建设，充分利用“庐山观光休闲旅游”品牌，积极探索资源共享、利益分成的经营机制，增强庐山核心景区的带动力。高起点编制云居山－－柘林湖国家级风景名胜区总体规划，整合云居山－柘林湖旅游资源，理顺管理体制，打造“云柘宗教文化山水游”品牌。整合市区文化旅游资源，规划建设龙开故道旅游带，开发保护浔阳楼、琵琶亭等历史文化景点，打造“浔阳古韵文化游”品牌。

开拓境外客源市场。充分发挥旅游营销中心和网络营销作用，组织直航旅游包机来九江旅游，巩固香港、澳门等传统客源市场，主攻韩国、台湾、新加坡和日本等新兴市场，拓展欧、美和澳洲等潜在市场。鼓励境外旅行社以股份或独资形式进驻九江，在境内外主要客源地设立九江旅游宣传营销窗口，逐步实行旅行商代理制，办好九江（庐山）国际旅游节。实现全年接待旅游人数突破千万人次，其中入境旅游者突破１０万人次。

加快旅游产品的开发建设。精心编排市区仿古文化名城游、庐山山水文化游、南线宗教文化山水游、东线名湖名洞游的精品线路。重点规划建设云居山－柘林湖景区、东林大佛、吴城候鸟观光、铜岭古矿冶遗址、鄱阳湖水上游等项目。建设旅游商品交易市场，加快珍珠饰品、剪纸艺术、特色食品等旅游商品开发。

（六）完善城市功能，加强城市管理，着力推进城市大建设

发挥规划先导作用。朝着建设１００万人口中心城市目标，制订好１００平方公里的控制性详规，完善１００平方公里的城市功能，培育支撑１００万人口的产业，今年重点推进１００个重大项目，力争完成１００亿元的投资，全力打造中心城市。组织完成九江城市未来２００∽３００年远景规划，力争详细规划覆盖率达９５％，都市圈内县城及中心镇的控规覆盖率达８０％，村庄规划编制率达６５％以上。完成城市防灾、旅游、山水生态保护等专项规划。做好环庐山周边协调发展规划和重要地区控制性和修建性详规。

加快完成重点城建工程。推进南湖市民文化广场、胜利碑及胜利公园、龙开故道、会议中心、体育中心等城市续建项目建设。启动建设博物馆、浔阳古城、艺术中心、江湖楼、鹤问湖污水处理厂、鹤问湖环湖大道、四水厂一期、第二垃圾处理场等项目。建设５万平方米的经济适用房和３万平方米的廉租房。

多渠道筹集城建资金。按照市场主导和政府引导相结合的原则，积极探索城市建设融资新途径，采用ＢＯＴ方式建设城市公共设施和基础设施。加大政府对土地一级市场的垄断，以地建城。全面实施污水处理、垃圾处理市场化运作，逐步将市政园林设施管护推向市场。各县（市、区）根据自身特色，选准突破口，加大投入，加快城镇建设。

进一步巩固城市整治成果。在去年狠抓城市整治工作的基础上，健全城市管理长效机制。维护城市规划的权威性和严肃性，提高城市执法效率，坚决制止和取缔各种违法建设行为。加强城管队伍建设，健全市、区、街道、社区相衔接，合理分工、规范高效的城市管理体系。加大城市管理综合执法力度，从严整治市容市貌和交通秩序。

（七）全面发展社会各项事业，努力构建和谐社会

以人为本，就业为先。完善公共就业体系，鼓励自主创业、自谋职业和非公有制经济、福利企业吸纳就业，不断增加就业总量和残疾人就业，及时调整最低工资标准。加快完善劳动力中心市场建设，为困难群体、大中专毕业生和被征地农民免费提供职业介绍、就业指导、职业培训等“一站式”服务。完善城镇职工基本养老、基本医疗和失业、工伤、生育保险制度，进一步扩大养老保险覆盖面和养老金社会化发放面，提高基金征缴率和备付能力。做好国有企业下岗职工基本生活保障与失业保险并轨工作。进一步完善城市最低生活保障制度、农村特困群众救助制度，建立农村医疗救助制度，提高农村“五保户”集中供养率，逐步建立与经济社会发展水平相适应、覆盖城乡的救助体系。继续开展创建全国双拥模范城“三连冠”和全国社区建设示范市活动。进一步构建公共财政体系，深化部门预算、国库集中支付、乡财县代管、村帐乡代理等财政改革，努力提高财政保障能力。

坚持把科教放在优先发展的战略位置。树立人才是第一资源观念，加快人才队伍建设，积极培养和引进急需的各类人才，完善人才竞争、激励、评价、分配机制。大力推进科技进步，增强技术创新能力，完善以企业为主体的技术创新体系和以学院、科研机构为主体的知识创新体系。加强科学普及工作，积极研发实用型技术和产品，加快科技成果向现实生产力转移步伐。统筹城乡教育事业，加大对农村义务教育投入，确保地震灾区学生新学年回到新校舍，今年解决全市中小学生自带课桌椅问题。完善“一费制”改革，建立“控辍保学”机制，确保困难家庭学生就学。积极推进职业教育和民办教育，切实加强高校建设，为经济建设提供人才支持。

繁荣和发展社会各项事业。加强精神文明建设，广泛开展“创新创业、科学发展”主题教育活动。抓好九江书城、九江影城建设，培植文化产业新的增长点。加强公共卫生建设，完善疾控体系和医疗救治体系，扩大新型农村合作医疗试点，尽快完成市县疾病控制中心、传染病医院（区）建设任务，继续做好血吸虫防治工作。加强医疗市场整顿，着力解决群众看病难、看病贵的难题。深入开展全民健身活动，提高竞技体育水平。积极开展计划生育优质服务，稳定低生育水平，促进优生优育，改善人口结构。做好外事侨务、民族宗教、妇女儿童、残疾人保障等工作，推动新闻出版、广播电视等各项事业的全面发展。

切实抓好安全生产和社会稳定工作。全面贯彻《中华人民共和国安全生产法》，进一步落实安全生产责任制和特、重大安全事故行政责任追究制度，强化监管，严格执法，专项整治，排查隐患，严防重大安全生产事故发生。加强各种自然灾害预测预报，提高防灾抗灾减灾能力。强化对食品、药品、餐饮卫生等行业的监管，保障人民群众健康安全。积极推进社会治安综合治理，完善社会治安防控体系，建立健全群体性事件预警机制、矛盾排查调处机制、突发公共事件应急机制。

（八）加强政府自身建设，提高执政为民的服务水平

围绕建设“开拓、透明、高效、诚信”政府的目标，把政府工作纳入制度化、法制化轨道，把落实和效率贯穿于每一个环节、每一个节点，使市委的思想力、决策力迅速、准确地转化为各级政府的执行力和操作力，不断提高政府的公信力。

提高执行力。面对新的发展形势，我们必须进一步加强对经济、科技、法律等知识的学习和掌握，提高对中央、省和市委方针、政策、决策的领悟能力，深入基层，加强调查研究，吃透上情增强执行的自觉性，掌握下情提高解决问题的实际能力。坚持坚韧性、开创性的统一，倡导“执行没有借口”的工作理念，对已明确的任务，确定的事项建立分工负责制、过程督查制、结果追问制，真正做到责任到人、奖惩兑现，坚决杜绝推诿扯皮。继续巩固“两个规范”教育成果，进一步加强行政机关效能建设，提高依法行政能力。

提高操作力。对重点工作的突破和影响全局的大事要一抓到底，倡导“凭结果说话”的业绩评价理念，加大督查工作力度，从大处着眼、小处着手，创造性地开展工作，切实提高为县（市、区）、为基层服务的水平。建立健全深入了解民情、充分反应民意、广泛集中民智、切实珍惜民力的政府决策机制，实行重大决策听证制度、公众参与制度和政务公开制度，完善专家咨询制度，确保政府决策的民主化、科学化。坚决贯彻落实市委各项决策，依法接受市人大及其常委会的监督，及时报告工作，认真落实各项决议、决定，积极支持人民政协履行政治协商、民主监督、参政议政的职能，主动听取各民主党派、工商联和各人民团体的建议和意见，认真办理人大代表议案和政协委员提案。

提高公信力。进一步规范事权、财权，强化审计监督，严格领导干部廉洁自律制度。以务实的作风、扎实的工作，继续多办、快办惠民之事，在完成好１００件惠民实事跨年度项目的同时，重点搞好“零就业”家庭的“帮扶工程”、维护社会稳定的“安宁工程”、促进可持续发展的“环保工程”、地震灾区的“重建工程”，以及政府带头的“关爱工程”，在实干中树立形象，在成事中赢得民心。加强社会信用体系建设，完善行政审批责任制和过错责任追究制，继续推行行政机关服务承诺制度。加快建立企业信用评价、监督管理、责任追究等信用制度，促进企业守法经营。重视广大群众和新闻媒体的监督作用，加强诚信教育，提高公民诚信意识，加大失信惩戒力度，依法维护社会公正。

各位代表！蓝图已经绘就，机遇催人奋进，使命就是责任。生逢盛世，当有所为。让我们高举邓小平理论伟大旗帜，努力实践“三个代表”重要思想，坚持以科学发展观统领全局，紧密团结在以胡锦涛同志为总书记的党中央周围，在省委、省政府和市委领导下，紧紧依靠全市人民，以百折不挠的坚强、心无旁鹜的信念、全力以赴的投入，励精图治，扎实工作，为建设美丽、富裕、和谐、强盛的社会主义新九江而共同努力！
